# Measuring gut perfusion and blood flow in neonates using ultrasound Doppler of the superior mesenteric artery: a narrative review

**DOI:** 10.3389/fped.2023.1154611

**Published:** 2023-08-02

**Authors:** C. Murphy, S. Baskind, N. Aladangady, J. Banerjee

**Affiliations:** ^1^ Neonatal Intensive Care Unit, Homerton Healthcare NHS Foundation Trust, London, United Kingdom; ^2^ Neonatal Intensive Care Unit, Queen Mary University of London, London, United Kingdom; ^3^ Neonatal Intensive Care Unit, Imperial College Healthcare NHS Trust, London, United Kingdom; ^4^ Neonatal Intensive Care Unit, Imperial College London, London, United Kingdom

**Keywords:** gut blood flow, gut perfusion, NEC, preterm infants, doppler ultrasound, superior mesenteric artery doppler

## Abstract

The gut is a relatively silent organ *in utero* but takes on a major role after birth for the absorption and digestion of feed for adequate nutrition and growth. The neonatal circulation undergoes a transition period after birth, and gut perfusion increases rapidly to satisfy the oxygen demand and consumption. If this process is compromised at any stage, preterm and fetal growth restricted infants are at particular risk of gut tissue injury secondary to hypoxia, leading to necrotizing enterocolitis. Feeding can also be a challenge in these high-risk groups due to gut dysmotility. Superior mesenteric artery (SMA) Doppler is a safe, bedside investigation that could rapidly aid clinicians with feeding strategies and in monitoring high-risk infants. This article aims to establish normal patterns of gut blood flow velocity in neonates using SMA Doppler and reviews how it might be used clinically in pathologic states.

## Introduction

Following birth, there is a transition from fetal to newborn circulation, and thereafter, a newborn grows rapidly. Consistent forward flow through the superior mesenteric artery (SMA) to the gut is needed to perfuse the gut in order to support the main gut function, which is the absorption of nutrients for adequate growth. SMA Doppler ultrasound (US) allows us to assess the direction and velocity of gut blood flow; these factors affect a neonate's tolerance to feed. It may also be helpful in predicting and identifying the onset of gut-related diseases, such as necrotizing enterocolitis (NEC), and may guide clinicians on the best timing of blood transfusion (BT) for anemia, as this is currently under debate.

## Basic principles of ultrasound

Doppler US has a high frequency and a short wavelength than the audible sound so it can be turned into a beam. A US probe emits a vertical beam and measures the returning echoes to formulate an image; if the echo is received from a moving tissue or cell (e.g., blood flow), then the transmitted and received frequencies are not equal. This is called the Doppler principle.

Blood flowing toward the probe has a higher frequency (red on color Doppler), and blood flowing away from the probe has a lower frequency (blue on color Doppler). Velocity, ultrasound frequency, and angle of insonation all influence the Doppler waveform. The higher the velocity, the higher the Doppler and US frequency; however, tissue penetration is better at lower US frequencies. A balance between the best achievable sensitivity to blood flow and tissue penetration is required. A pulsed wave Doppler (PWD) emits a single wave repeatedly fired at a particular frequency called pulse repetition frequency (PRF), and the echoes received from the moving blood cells will shift in frequency, which determines the blood velocity. PRF is also referred to as a scale on the echo machine. This is important where the blood velocity is not uniform [limited by changes in direction or at the level of valves/shunts such as patent ductus arteriosus (PDA)]. The highest detectable velocity is half the PRF (known as the Nyquist limit). PWD use is limited by aliasing where the blood velocity and beam/flow angle are greater than the Nyquist limit, which causes ambiguity with the results. For example, aliasing may occur if a low scale (low PRF) is used on a vessel with high velocity. This may cause the direction of blood flow to be registered incorrectly (known as color aliasing artifact). To prevent aliasing, the scale should be increased (which increases PRF) and the angle of insonation adjusted.

## Ultrasound and Doppler use in medicine

US is widely used in many aspects of medicine and is useful for screening and prevention (e.g., mammograms), in diagnosis and assessing anatomy, and in assisting procedures (such as vessel catheterization). Doppler US produces flow velocity waveforms that allows physicians to monitor and identify changes in organ perfusion.

The peak systolic velocity (PSV), end diastolic velocity (EDV), and time-averaged mean velocity (TAMV) are used to calculate the pulsatility index (PI) and resistance index (RI) (1). See formula below:

PI=(PSV−EDV)/TAMVRI=(PSV−EDV)/PSV

PI and RI are related measures of arterial pulsatility and can identify resistance in the blood vessel. The mean blood flow velocity (BFV) is often used to describe organ perfusion, and Doppler indices add information regarding pattern evolution over time.

Assessing the velocity of blood flow to a tissue alone does not directly describe the oxygenation of that tissue. Several other factors affect the blood's affinity for oxygen and its release to tissues (such as temperature, degree of acidosis, and proportion of fetal hemoglobin), which is well described by the oxyhemoglobin dissociation curve and Bohr effect.

## Ultrasound and Doppler use in neonates

US is a safe and convenient bedside investigation and widely used in neonates in both clinical and research settings. Clinically, it is used regularly for the monitoring of the brain and is a valid bedside test to exclude intraventricular hemorrhage and parenchymal changes. US and Doppler US are also used to assess the neonatal skeleton, heart, abdomen, kidneys, and liver.

## Gut blood flow and Dopplers

The primitive gut (made up of fore, mid, and hind gut) is supplied by three anterior aortic abdominal arteries. The coeliac trunk supplies the foregut (esophagus, stomach, and proximal duodenum as well as the hepatobiliary structures, pancreas, and spleen); the SMA supplies the midgut (the duodenum distal to ampulla of Vater, jejunum, and ileum; ascending colon and proximal two-thirds of the transverse colon); and the inferior mesenteric artery supplies the hindgut (distal third of the transverse colon, descending colon, and rectum) (2).

*In utero* nutrients pass via the placenta from the mother to the fetus, and the gut vasculature is relatively inactive. Achiron et al. investigated the changes in fetal SMA flow from 14 to 37 weeks gestation in normal fetuses; as expected, the intestinal resistance remained high throughout the pregnancy (as indicated by PI) (3).

Gut oxygen demand changes dramatically after birth. The relatively silent organ *in utero* requires a rapid switch to immediately absorb and digest nutrients for an animal to grow. Animal and human studies suggest that human gut maturation starts earlier in fetal life but is slower than other mammals postnatally (well beyond the age of weaning) (4, 5). This could be impacted by preterm birth where the gut and lungs are immature. The fetus comes from a relatively hypoxic environment *in utero*, and a rapid and dramatic rise of arterial oxygenation is seen when breathing is established. If this does not occur (e.g., due to immature preterm lungs/birth asphyxia), then the gut does not function as expected [which explains the increased risk of NEC seen in preterm and fetal growth restriction (FGR) infants] (5). As the SMA irrigates the majority of the gut of interest in neonatology, few studies have interrogated the SMA blood flow velocities delineating the effects of various factors such as growth restriction and prematurity (Table 1).

**Table 1:** Factors affecting PSV in the SMA.

Factors that increase PSV	Factors that decrease PSV
Rising postnatal age	Early postnatal age
Anemia (low hematocrit)	Blood transfusion (high hematocrit)
Higher feed volume	Low feed volume/nil by mouth
Early commencing of feed	Late commencing of feed
No respiratory support	Respiratory support: CPAP/ventilation
Closed PDA	Large PDA
	Sepsis

PSV, peak systolic velocity; SMA, superior mesenteric artery; PDA, patent ductus arteriosus; CPAP, continuous positive airway pressure.

## Gut disease in neonates

Gut disease in neonates is a particular challenge as we rely upon radiographic and laboratory findings to assess likelihood of diagnosis. However, many of these findings are not specific to that pathology, rather they appear in multiple gut pathologies. NEC is a potentially devastating inflammatory bowel condition, which affects mainly preterm infants (up to 10%) (6); however, the incidence differs depending on gestation, birth weight, and country at birth (7).

Gordon described that the diagnostic “pitfall” surrounding NEC is the inclusion of “NEC-like” illnesses in NEC data sets (8). For example, clinical signs of spontaneous intestinal perforation (SIP) are akin to the presentation of NEC. Often, diagnosis of SIP is only made surgically, so these infants may be incorrectly included as NEC if surgery is not warranted. Pneumatosis is pathognomonic for NEC, but is only present in 40% of the cases (9). Much work is being done to tighten NEC diagnosis, and clinically the focus lies on reducing the risk of NEC using feeding strategies. Currently, clinicians err on the side of caution if there are any abdominal concerns and stop feeding/start antibiotics, which are not conducive to good weight gain or beneficial to the gut microbiome (10).

## Effect of anemia and blood transfusion on gut blood flow

Up to 90% of extremely preterm infants require a BT to treat anemia in the neonatal unit. However, evidence for treating anemia in infants is conflicting; both anemia and BT may play a part in the development of gut tissue injury and NEC, possibly due to a period of gut hypoxia followed by a reperfusion injury. In addition, multiple studies suggest a causal relationship between BT and NEC [known as “transfusion-related NEC” (TRNEC)]. This relationship has been identified as a priority area for international researchers, and a recent meta-analysis of liberal and restrictive BT approaches showed no difference in the incidence of gut tissue injury (11).

SMA Doppler US may assist in identifying those infants who have reduced gut blood flow due to anemia. The basal intestinal vascular resistance drops rapidly after birth, and gut blood flow increases significantly during the first week of life (12). Several factors may impair this transition including anemia (13), need for cardiorespiratory support, and use of parenteral nutrition (12). Our group performed SMA Dopplers pre- and post-transfusion in 59 preterm infants and found higher pre-transfusion SMA PSV in babies ≥8 days of age compared with those who were transfused in the first week of life; however, there were no changes in SMA PSV or EDV following BT (14). Several studies have demonstrated an attenuated response to feed immediately after BT, and this may last for 24–48 h (15). Currently, it is also not clear whether to withhold feeds or not during BT and the international WHEAT trial (WithHolding Enteral feeds Around packed red cell Transfusion to prevent necrotizing enterocolitis in preterm neonates) is likely to answer to this uncertainty (16).

## Assessing intestinal blood flow

Performing a duplex scan of the mesenteric vessels was first suggested by Nicholls in the mid-1980s to aid the diagnosis of chronic intestinal ischemia in adults (17). Since the 1990s, the patterns of flow in SMA Doppler were established and deemed as highly accurate in detecting SMA stenosis/occlusion with a diagnostic accuracy of up to 90% (18–21). By catheterizing the SMA and using dye dilution in adults, Norryd et al. showed a redistribution in blood flow after a meal and a corresponding drop in vascular resistance (22). Celiac artery flow did not demonstrate change in response to feed and did not affect SMA flow in any studies (17, 22, 23).

## SMA Doppler in neonates: validity and what is already known

Cardiac output is distributed approximately equally to the upper and lower parts of the body in a healthy newborn (14), with abdominal organs accounting for the largest proportion of blood flow to the lower part of the body. After birth, there is a dramatic increase in intestinal growth and oxygen demand, and the SMA BFV increases significantly in the first few weeks of life to support this, particularly in preterm infants where the changes are more rapid. Where this rapid rise is not seen, infants are more likely to have intestinal dysmotility and feed intolerance (24, 25).

Various studies have measured the SMA blood flow in clinically stable neonates (Table 2). Stritzke et al. studied 21 healthy neonates born >36 weeks gestation to characterize brain, gut, and kidney BFV over time during the first 24 h. SMA PI and RI was elevated at birth and dropped significantly over time; it had the highest blood flow and Vmax and the largest variation compared with the other major arteries. The reported Vmax of 70.6 cm/s in SMA at 24 h corresponds with other reported normal values between 38 cm/s and 90 cm/s, and the wide variation is also similar to previous findings (probably related to feeds) (26, 27). These findings support the concept of an independent autoregulation of blood flow in different organs, and the greatest change in SMA occurred in the first 2 h after birth (27, 28). The low resistance, high blood flow pattern is similar to the studies using near-infrared spectroscopy (NIRS) to assess gut oxygenation (29).

**Table 2:** Studies using SMA Doppler to assess gut blood flow in clinically stable neonates: validation of SMA Doppler in neonates.

Validation of SMA Doppler in neonates				
Author and year	Population and design	Intervention	Outcome	Limitations
Stritzke et al. (2019)	Single-center observational study of 21 infants.	SMA Doppler performed at three time points to characterize pattern of transition after birth:	↓PI and RI over time	Studied infants for 24 h only. Likely transitioning was not complete at the end of study. Not all measurements done by one operator.
			Greatest changes seen in the first 2 h after birth.	
	Inclusion: ≥36 weeks’ gestation.			
		<2 h of life		
	Exclusion: congenital anomaly, palliative condition, or factors known to prolong transition.	2–6 h of life		
		24 h of life.		
				Did not take feed status into account.
				50% received delayed cord clamping.
Bel et al. (1990)	Prospective single-center study of 128 infants.	SMA Doppler performed immediately pre-feed to establish normal values (PSV, EDV, TAMV, and PI calculated).	↑PSV, EDV and TAMV *α* ↑weight and ↑GA	13 infants SMA not visualized so measurements not taken.
	Inclusion: SGA, AGA, term and preterm infants.		Infants with PDA: ↓/reversed EDV	
				Further 6 infants were excluded because of issues with intestinal gas and restlessness.
			SGA vs. AGA infants: Different waveforms in SGA for 3 days: more pronounced positive BFV during diastole and ↑EDV on Day 1	
	Term infants with coarctation (*n* = 3).			
	Exclusion: not stated.			
			↓ PI in AGA babies on Day 1 and 2	
			All infants with coarctation of aorta:↓PSV and EDV, and ↓PI	
Martinussen et al. (1994)	Prospective study of 20 infants.	Performed SMA Doppler (PSV EDV, and TAMV).	Group 1	Small sample size.
			↓TAMV between 1 h and 6 h, ↑TAMV to baseline by 24 h	
	Inclusion: healthy term infants, AGA, normal vaginal delivery after uncomplicated pregnancy.			Only investigated Day 1–5.
		Group 1: 3 time points		
			EDV low at birth and ↑EDV over time.	
		<24 h of life: pre-first feed (20–50 min after birth); 6 h and 24 h of life.		
			Group 2:	
			Pre-feed: ↑TAMV, ↑EDV, and ↓resistance from Day 2–3 then plateau.	
	Exclusion: FGR infants who had already been fed.			
		Group 2: 4 time points		
			Post-feed: ↑TAMV, ↑EDV, and ↓resistance	
		2 h of life; and pre- and post-feed on Day 3, 4, and5.		
			Volume of feed did not affect post-feed response.	
Havranek et al. (2006)	Prospective study 27 infants.	Daily fasted/pre-prandial SMA PSV and TAMV from Day 1–5 of life to identify clinical variables that relate to postnatal increase in gut BFV.	All infants: ↑PSV and TAMV Mean PSV ↑ = 4.4 cm/s/day (*p* < 0.001).	Small sample size
			Mean TAMV ↑ = 0.51 cm/s/day (*p* = 0.005). Postnatal age α PSV (*p* = 0.037).	
	Inclusion: <34 weeks gestation, <2,500 g, AGA.			
	Exclusion: major congenital anomalies; cardiac abnormalities (including PDA); anemia (Hb < 10 g/dl) or polycythemia (Hb >22 g/dl); need for inotropic support, ventilation, or umbilical artery catheters.			
			Total volume of enteral feeds α TAMV (*p* = 0.018). Infants requiring CPAP >3 days /PN >3 days had attenuated increase in TAMV and PSV.	
Gillam-Krakauer et al. (2013)	Prospective single-center study of 25 infants.	Abdominal NIRS (A-rSO_2_) performed continuously for 72 h from enrollment.	PSV, EDV, and TAMV α toA-rSO_2_ pre-feed and 60–120 min post-feed.	Small sample size.
				Only provided 3 days of measurements in the first 2 weeks of life.
	Inclusion: healthy infants 23–30^+6^/40, <1,500 g, ≤14 days of age and tolerating enteral feeds of 10–20 ml/kg/day at start of study.			
			No correlation between SMA Doppler and A-rSO2 changes 10 min post-feed.	
		SMA Doppler performed 3 times per day on 3 consecutive days (each set of 3 occurred immediately pre-, 10 min post-, and 60–120 min post-feed).		
	Exclusion: congenital malformations, chromosomal anomalies, FGR, GI anomalies, previous NEC, need for inotropic support within 24 h, renal anomalies, or feed intolerance within 12 h of enrollment.			
		All infants were bolus fed.		

SMA, superior mesenteric artery; PSV, peak systolic velocity; EDV, end diastolic velocity; PI, pulsatility index; RI, resistance index; TAMV, time-averaged mean velocity; FGR, fetal growth restriction; SGA, small for gestational age; AGA, appropriately grown for gestational age; GA, gestational age; GI, gastrointestinal; PN, parenteral nutrition; NIRS, near-infrared spectroscopy; NEC, necrotizing enterocolitis; PDA, patent ductus arteriosus; A-rSO_2_, abdominal regional saturation.

Several factors impact intestinal blood flow, both intrinsic (including the cardiovascular status of the baby, mural and humoral control of vasculature) and extrinsic (volume and type of feed and certain drugs). It is important to optimize the factors we can control and understand better how they affect the rate of rise in SMA BFV especially in preterm infants (24). Research suggests that gestational age (GA), weight, FGR, feed, presence of PDA, and cardiovascular abnormalities all influence SMA BFV (30); and neonatal Dopplers correlate with disease severity and may differentiate those with anemia (27, 31, 32). The effects of these clinical factors will be individually discussed in more detail.

## Influence of gut ischemia, gut tissue injury, and NEC on SMA Doppler

Compromising blood flow to the intestines causes hypoxia and ischemia. The cause of NEC is multifactorial, but perfusion and oxygenation of the gut certainly plays a part. Researchers have studied the effect of gut pathologies on the SMA blood flow (Table 3). Murdoch et al. postulated that SMA Doppler performed on day 1 may identify infants at risk of NEC and dysmotility. They found that preterm infants with abnormal antenatal Dopplers who later developed NEC were more likely to have a raised PI on day 1 of life, suggesting that abnormal splanchnic circulation in fetal life or vasoconstriction in neonatal life increases the risk of NEC and this could be identified using SMA Doppler (33). However, this was not supported by a more recent study who found SMA Doppler on day 1 of life in preterm FGR infants to be a poor predictor of NEC (34).

**Table 3:** Studies using SMA Doppler to assess gut blood flow in times of pathology: anemia, blood transfusion, sepsis, and NEC.

Author and year	Population and design	Intervention	Outcome	Limitations
SMA Doppler in relation to anemia and blood transfusion				
Banerjee et al. (2016)	Prospective single-center study of 59 preterm infants.	SMA Doppler measurements performed 30–60 min pre- and post-BT to measure changes in gut blood flow following BT.	Pre-BT PSV: G3 > G1 (*p* < 0.01) and G2 > G1 (*p* = 0.09).	Small sample size.
	Grouped by postnatal age:			
			PSV ↓ post-BT (trend) =	
	Group 1: 1–7days		Mean Hb pre-BT = 10 g/L.	
	Group 2: 8–28 days		No infant developed NEC in either group.	
	Group 3: ≥28 days.			
	Inclusion: 23–34-week gestation requiring BT.			
	Exclusion: major congenital abnormality; too unwell to handle.			
Krimmel et al. (2009)	Prospective RCT single-center study of 22 infants.	SMA Doppler performed 4 times for each infant:	Response to feed (during anemia) ↑PSV (*p* < 0.02) and ↑mean BFV (*p* < 0.01).	Only measured 30 min post-BT.
	Grouped by weight (<1,250 g or >1,250 g) and randomized to feed or fast during BT.	1: 30 min before the last feed pre-BT;		Underpowered to investigate NEC.
		2: 30 min after the last feed during anemia	No changes seen post-BT.	
	Inclusion: born at 25–32 weeks GA, ≤38 weeks at time of BT and receiving bolus enteral feed of at least 60 ml/kg/d.			
		3: 30 min post-BT and prior to next feed;		
		4: 30 min after completion of feed post-BT.		
	Exclusion: congenital and chromosomal abnormalities, TTTS, previous NEC, concurrent treatment for sepsis, feed intolerance, FGR and PDA.			
Pitzele et al. (2015)	Prospective single-center study of 25 infants.	SMA BFV (PSV, EDV, and TAMV) performed at 6 time points:	Significant ↑TAMV and ↑PSV post-feed during anemia (pre-BT) and 24 h post-BT.	Did not consider feed volume.
	Inclusion: ≤1,500 g, AGA, ≤36 weeks CGA at time of transfusion; tolerating ≥20 ml/kg/d in every 3 h bolus feeds.			Majority had small PDA that may explain lack of association.
		45 min pre- and post-feed	Presence of PDA did not affect SMA BFV.	
		Immediately pre- and post-BT		
		Pre- and post-feed measurements repeated at 24 h and 48 h.		
	Exclusion: multiple pregnancy, major congenital and chromosomal anomalies, CHD, shock, on inotropes, previous NEC.			
SMA Doppler in relation to sepsis				
Kempley et al. (2014)	Prospective dual-center study of 76 infants.	SMA Doppler performed during the first 24 h of life (PSV, EDV, and PI)	Infants with culture-positive sepsis (Group 4) had ↓PI (significant) and ↑BFV (trend) compared with infants with no positive culture (groups 1, 2, and 3);	Small sample size.
	Inclusion: <37 weeks GA, results of initial cultures available, BW >10th centile.			
		Aim: to determine the effect of perinatal bacterial infection on the gut circulation.		
				Wide variation in degree of prematurity “<37 weeks.”
	Exclusion: SGA infants.	For analysis, combined groups 1, 2, and 3: “no positive culture vs. Group 4 “culture-positive sepsis”.		
	Group1: negative culture no PROM			
	Group 2: PROM with negative culture;			
	Group 3: gastric aspirate, ear swab or umbilical swab positive with negative blood culture; Group 4: positive blood cultures.			
Hashem et al. (2017)	Prospective study of 51 infants. Inclusion: <37 weeks with neonatal sepsis.	SMA Doppler performed for PSV EDV and PI and RI calculated.	↑PSV and EDV in group 1 vs. Group 2 (*p* < 0.001).	Small sample size. Included infants with feed intolerance in NEC group.
	Exclusion: congenital malformations, unstable hemodynamically, UAC or previous abdominal surgery.			
	Group 1: with abdominal signs of feed intolerance or NEC			
	Group 2: no NEC.			
SMA Doppler in relation to NEC				
Louis et al. (2013)	Prospective single-center study. 100 infants:	Performed SMA Doppler on Day 1 and 5 of life (PSV, EDV, PI, RI) Does SMA Doppler predict risk of NEC in IUGR infants?	Baseline SMA Doppler similar between groups	No AGA control group. Underpowered to look at prediction of NEC.
	Group 1: 50 IUGR + A/REDF			
			NEC incidence Group 1 > Group 2 [32% vs. 4% (*p* < 0.01)].	
	Group 2: 50 IUGR + normal Doppler (control).	Prospectively recorded babies who later developed NEC based on diagnosis by medical team.		
	Inclusion: <36 weeks with IUGR and A/REDF or normal Dopplers.			
			Infants who developed NEC: Group 1 RI on Day 1 (5.4 (IQR 3.3–7.3)> Group 2 RI on Day 1 (3.3 (IQR 1.7–3.9) (*p* = 0.049).	
	Exclusion: major congenital malformations, perinatal asphyxia, shock, congenital heart disease.			
			In Group 1: Day 1 EDV and Day 5 PSV in infants with NEC < infants without NEC (*p* = 0.03; *p* = 0.02).	
			RI on Day 1 sensitivity 61%, specificity 57% for predicting NEC.	
Murdoch et al. (2006)	Prospective single-center study of 64 infants.	SMA Doppler performed on Day 1 of life when hemodynamically stable. (PSV, EDV, RI, and PI).	↑ NEC in infants with ↓EDV and ↑PI.	Used both suspected and confirmed cases of NEC.
	Inclusion: <24 h of life at study entry.		As risk of NEC↑ -> TAMV↓.	
		Clinicians were blinded to Doppler results.	As GA ↑ -> ↑PI and ↓EDV.	
	Exclusion: congenital anomalies or proven sepsis			
		Infants were monitored for NEC to see if SMA Doppler on Day 1 can predict NEC.		

SMA, superior mesenteric artery; PSV, peak systolic velocity; EDV, end diastolic velocity; EDF, end diastolic flow; A/REDF, absent/reversed end diastolic flow; PI, pulsatility index; RI, resistance index; TAMV, time-averaged mean velocity; BFV, blood flow velocity; FGR, fetal growth restriction; SGA, small for gestational age; AGA, appropriately grown for gestational age; GA, gestational age; CGA, corrected gestational age; PROM, prolonged rupture of membrane; NEC, necrotizing enterocolitis; PDA, patent ductus arteriosus; CHD, congenital heart disease; BT, blood transfusion.

## Limitations of SMA Doppler

The normal pattern of gut blood flow is more difficult to establish than of the other organs as it is a motile organ and variation in intestinal wall tension affects vascular resistance inversely. The GI tract produces hormones that dilate intestinal vessels in response to enteric feed, which also contributes to increased blood flow (14).

Doppler assessment requires trained personnel and is prone to operator-dependent bias. The internal diameter of the vessel affects results and needs to be accurately measured (26). Neonatal blood vessels are <3 mm in diameter, bordering on the limit of resolution due to their size; fortunately, this is now less of a challenge with the availability of the more sophisticated color Doppler equipment (26). The angle of insonation affects results; Coombs et al. described an error of 10% in velocity for a 5% angle error when studying SMA flow, while Bel et al. found that an insonation angle of >15° caused underestimation in velocity (30).

Doppler provides a snapshot in time and does not allow continuous evaluation and does not provide a direct estimate of regional tissue perfusion (35). Other studies have reported difficulties with intestinal gas obscuring the view, infant restlessness, and variations in the course of proximal SMA that led to an unfavorable insonation angle (30).

## Use of coeliac trunk Doppler in neonates

NEC is least likely to occur in the portion of gut supplied by the coeliac artery. There is no proven correlation between the coeliac artery and SMA Doppler results in infants with NEC (17, 25, 26, 33). Some studies suggest a “qualitatively similar but quantitatively smaller” response to feed compared with SMA Doppler (26) but a significant reduction in the flow volume of the celiac artery has been demonstrated following blood transfusion (36). However, coeliac trunk Doppler to assess gut blood flow in relation to gut tissue injury is unlikely to be clinically useful in neonates.

## Clinical implications of Doppler flow

### Effects of GA and postnatal age on SMA Doppler

Bel et al. studied normal values for SMA BFV in 128 preterm and term infants and identified a positive correlation between rising BFV (all parameters) with GA and body weight (30); this was similar to Murdoch et al. (33). Resistance (as evidenced by PI) was higher in lower gestations suggesting that degree of prematurity is associated with higher mesenteric flow resistance (33).

Our group studied infants in three postnatal age groups in the context of blood transfusion and found a significant positive correlation between pre-transfusion PSV and postnatal age; this difference was particularly apparent when comparing infants in the first week of life vs. >28 days. This was attributed to the circulatory adaption after birth and possibly the presence of PDA. However, higher PSV with postnatal age remained significant when corrected for GA, birthweight (BW), and the presence of PDA (37).

### Effects of FGR and SGA on SMA Doppler

Fetal brain sparing or cardiovascular adaptation that occurs during FGR is to the detriment of the “non-essential” fetal organs such as the gut and is particularly evident where abnormal umbilical Doppler results are present (38, 39). Infants predisposed to gut hypoxia develops feed intolerance and therefore are at increased risk of developing NEC (38, 40–43). These infants are also more likely to require BT for anemia (44).

Several studies have demonstrated a positive correlation between gut BFV and body weight (30, 33, 45). FGR infants appear to have different waveforms compared with their appropriately grown for gestational age (AGA) counterparts (30). Infants with FGR have high resistance patterns of flow in the first few days of life particularly if they later develop NEC (34), suggesting that *in utero* resistance/vasoconstriction persists after birth (33).

### Effects of anemia and BT on SMA Doppler

In many forms of anemia, the hematocrit is low causing reduced viscosity and increased velocity, and our group have demonstrated higher SMA PSV in anemic preterm infants during the first week of life (46). Anemia may not impair post-prandial increase in mesenteric blood flow but causes a greater than normal increase in velocity in response to feed (47). Blood viscosity increases following BT (up to 20%), and as expected, several studies have demonstrated a concurrent significant reduction in the mean PSV (15, 46, 47). However, it is not clear how long these changes persist for after BT (15). The effects of anemia and blood transfusion on SMA Doppler are summarized in Table 3.

### Effects of sepsis on SMA Doppler

In the septic state, acute hypotensive shock may occur, and vascular bed arterioles constrict in response to pathologic decrease in systemic vascular resistance. Redistribution occurs that reduces splanchnic flow, predisposing infants to gut tissue hypoxia and injury as early as day 1 of life (48). Animal and human models suggest a rise in RI, and a drop in EDV is seen in culture-positive sepsis (49).

The risk of NEC increases with raised PI and falls with high EDV, and infants with risk factors for NEC tend to have higher resistance patterns as early as day 1 of life (48). Studies using SMA Doppler during NEC are outlined in Table 3. However, in active NEC/sepsis, this resistance reduces due to dilatation of the vascular bed and inflammation (26, 33).

### Effects of feeding on SMA Doppler

Feeding is often difficult in preterm and FGR infants, and clinicians approach cautiously (43). It is the introduction of colostrum after birth that increases gut blood supply and encourages development of vascular bed. Human gut maturation starts early in fetal life, but is slower than other mammals and does not fully mature until beyond the time of weaning onto solid foods (5). Both animal and human models demonstrate a rapid rise in SMA BFV and a drop in vascular resistance on Doppler in the first week of life as feeding is established (5, 45, 50). This is clinically significant as a more rapid rise in SMA BFV reduces gut dysmotility, and a high resistance, reversed or turbulent flow pattern across SMA, is associated with a delay in establishing feeds (5, 50).

In normal infants, enteral feed stimulates intestinal motility and release of circulating vasoactive substance (e.g., CCK P2, secretin, and gastrin), which increases intestinal blood flow. It is accepted that PSV is higher in fed babies, and PSV rises further following a feed. Preterm infants are able to regulate SMA BFV in response to milk feeds, and increases in velocity have been shown to be positively correlated with feeding tolerance (26, 27, 52). Response may also be affected by the feed volume, frequency, and type (11, 25, 50). Havranek et al. demonstrated that the total volume of enteral feed (regardless of human or formula milk) was significantly related to a daily increase in TAMV (12). Lane et al. showed persistent hyperemic state in infants fed by hourly bolus compared with every 3 h bolus feeds with significantly higher pre-prandial PSV (70 vs. 53 cm/s). Those receiving breast milk had a longer but smaller change in amplitude compared with those receiving preterm formula (51), suggesting hourly bolus feeds cause persistent hyperemic state, and the composition of feed and feed interval affects gut perfusion (26, 51). Coombs et al. made similar conclusions with regard to bolus feeds that may persist for up to 2 h (26). Hypoxia/ischemia of intestine may alter the interaction between intestinal motility and the release of vasoactive substances in response to feed since infants who showed poor vasomotor response were symptomatic of gut dysmotility, and almost 50% of these infants went on to develop sepsis (25). This is outlined in more detail in Table 4.

**Table 4:** Studies using SMA Doppler to assess gut blood flow in the presence of confounding variables: effect of neonatal feeding and PDA.

Author and year	Population and design	Intervention	Outcome	Limitations
SMA Doppler and neonatal feeding				
Fang et al. (2001)	Prospective blinded single-center study of 56 infants.	SMA Doppler performed to see effect of first “test” feed on gut BFV (0.5 ml breast or formula milk via NGT).	Early tolerance to feed in infants with ↓RI at 60 min post-test feed (*p* < 0.0001).	First test feed occurred up to 30 days of life (some infants far beyond postnatal transition at this time).
	Infants divided into two:			
	Group 1 (*n* = 14): early tolerance to full feeds (within 7 days)		TAMV↑ post-feed	
		5 time points:	↑TAMV at 60 min post-feed	
			TAMV 1/α to number of days taken to tolerate feeds (*p* < 0.01). Poor response to feed ↑ likelihood of feed intolerance.	Large variation in GA (up to 36 weeks) and not assessed for effect of GA.
		Immediately pre-feed; and at 15, 30, 45, and 60 min post-feed.		
	Group 2 (*n* = 30): late tolerance (time to full feed >7 days).			
	Inclusion: <36 weeks gestation; without abdominal symptoms and prior to first feed.			
			SGA did not affect response to feed.	
	Exclusion: infants with umbilical catheters.			
Coombs (1992)	Prospective single-center study of 14 infants.	Daily SMA Doppler performed immediately pre- and post-feed from Day 1–5 of life to measure effect of fasted state and feed on gut BFV.	↑Mean fasting PSV, EDV and TAMV with postnatal age: significant Day 1–2 with daily upward trend until Day 5. Large ↑PSV, ↑EDV, and ↑TAMV post-feed. No correlation between Doppler and birth weight or gender.	Small study.
				Did not account for volume of feed (could explain difference between breast- and bottle-fed infants).
	Inclusion: term infants born via elective section.			
	Exclusion: infants requiring NG feeds.			
Lane et al. (1998)	Prospective single-center study of 62 infants.	SMA Doppler performed at 10 min intervals from 20 min pre-feed and for 60 min (if fed hourly), 90 min (if fed 2 hourly), or 120 min (if fed 3 hourly) post-feed.	Pre-feed ↓PSV in 3 hourly fed group vs. 1 hourly group (*p* < 0.01).	Small sample size.
	Inclusion: “first few days of life.”			
			Breast milk had a longer (42 vs. 27 min *p* < 0.05) but smaller (31 vs. 25 *p* < 0.05) change in amplitude vs. formula milk.	
			Feed volume did not show effect.	
Robel-Tillig et al. (2004)	Prospective single-center study of 478 infants.	SMA Doppler performed daily Day 1–5 of life (PSV, EDV, PI).	Infants with pathological parameters had significantly more intestinal dysmotility than in normal group (83% vs. 15%). Infants with normal SMA Dopplers were able to tolerate significantly more feed by Day 5. Strong negative correlation between pathological PI on Day 1 and quantity of tolerated feed on Day 5.	Most of the infants with dysmotility were SGA. Did not evaluate AGA vs. SGA.
	Inclusion: preterm with BW <1,500 g. Mean GA: 31.4 (Group 1), 28.6 (Group 2).			
		Infants divided into 2 groups:		
		Group 1: pathological blood flow (148 infants)		
		Group 2: normal blood flow (330 neonates)		
Maruyama et al. (1999)	Prospective single-center study of 44 infants.	Fasted or pre-feed SMA Doppler performed daily Day 1–6. All had started enteral feeds by Day 2: either 2 or 3 hourly.	↑BFV and ↓RI with postnatal age PDA -> ↓EDV on Day 1 ↑TAMV α with ↑ birth weight and number of enteral feeds	Stable preterm infants only in first week of life.
	Inclusion: AGA infants born <34 weeks who were otherwise well. Exclusion: ventilated; >30% FiO2; on inotropes; chromosomal abnormality or congenital disorder.			
		All infants had spontaneous closure of PDA by Day 3.		
SMA Doppler in relation to PDA				
Coombs et al. (1990)	Prospective single-center study of 37 preterm infants with and without PDA.	Group 1: bolus given over 20 s on 3 occasions 12 hourly.	Pre-infusion Absent/reversed EDF seen in all Group 1 and 2 infants.	Small study.
	Infants divided into three groups:	Group 2: slow infusion given over 35 min.		
			Group 3 infants all had forward flow (>Day 1 of life)	
				No infants developed NEC following bolus of indomethacin.
			Post-infusion	
			Group 1: significant ↓mean PSV (74–38 cm/sec). PSV took longer to recover in infants whose duct closed post-treatment. Group 2: fall in SMA ↓mean PSV (not significant).	
	Group 1: (*n* = 9) With PDA and receiving indomethacin by bolus;			
		SMA Doppler performed in fasted state, pre- and up to 2 h post-infusion to calculate the time taken to reach maximal change in velocity.		
	Group 2: (*n* = 10) with PDA and indomethacin by slow infusion			
	Group 3: without PDA (*n* = 18).			
		Aimed to investigate GI side effects of indomethacin (e.g., NEC).		
Hsu et al. (2020)	Prospective dual-center study of 25 infants.	Daily MCA, Renal, SMA, and coeliac artery Doppler performed Day 1–7 of life.	PDA-> ↑abnormal organ blood flow (39% vs. 8% *p* < 0.01).	Small sample size.
				Did not investigate beyond first week of life.
	Inclusion: inborn infants <30 weeks.		Size of PDA α resistance and 1/α velocity.	
		Abnormal organ blood flow = abnormal indices in ≥2 organs on same day of life or ≥2 abnormal blood flow on different days of life		
	Exclusion: chromosomal or major congenital anomaly, complex congenital heart disease and perinatal asphyxia, severe IVH/SIP/NEC/ culture-positive sepsis in first week of life. Abnormal blood flow considered as reversed/absent EDF, low mean/systolic velocities, and high PI/RI.		PDA ≥2 mm -> 8-fold ↑ in RI.	
Yanowitz et al. (2014)	Multicenter RCT (taken from parent study),	SMA Doppler performed at three time points:	No significant difference between PDA treatment groups.	Small sample size.
				90% of infants received indomethacin due to drug shortage.
	34 infants randomized to two PDA treatment groups:	18–24 h after drug treatment 10 min post 4 ml/kg test feed,	No significant difference between groups at baseline or post-feed.	
	Treatment 1: ibuprofen	30 min post 4 ml/kg test feed.	At 30 min post test feed: significant ↑PSV, ↑EDV and ↑mean BFV in both groups. Earlier ↑PSV and mean BFV in Feed Group.	
	Treatment 2: indomethacin			
	And then to either feed/fast during treatment:			
	Feed group: fed 15 ml/kg/d during PDA treatment			
	NBM group: NBM during treatment.			
	Inclusion: infants requiring treatment for PDA, <31 weeks gestation, 401–1,250 g.			
	Exclusion: infants receiving >60 ml/kg/day of feed prior to study entry.			

SMA, superior mesenteric artery; PSV, peak systolic velocity; EDV, end diastolic velocity; EDF, end diastolic flow; PI, pulsatility index; RI, resistance index; TAMV, time-averaged mean velocity; BFV, blood flow velocity; SGA, small for gestational age; AGA, appropriately grown for gestational age; GA, gestational age; GI, gastrointestinal; NG, nasogastric; NEC, necrotizing enterocolitis; PDA, patent ductus arteriosus; IVH, intraventricular hemorrhage; SIP, spontaneous intestinal perforation; NBM, nil by mouth; MCA, middle cerebral artery.

### Effects of PDA on SMA Doppler

The presence of a PDA is inversely related to gestational age, and while exact numbers differ, PDA is present in at least 50% of infants born at <28 weeks (52) but may be as high as 90% in <24 week infants and 80% in 25–28 weeks (53). A significant proportion of these is hemodynamically significant PDA (hsPDA) and requires treatment for closure (53, 54). An hsPDA is when a left to right shunt leading to pulmonary overcirculation and a left heart volume overload are seen. Clinically, infants might present with ventilator dependence due to pulmonary edema and ineffective gas exchange. An hsPDA and the resultant ductal steal lead to retrograde diastolic flow in the abdominal aorta, and a low antegrade or retrograde diastolic flow in systemic arteries leading to systemic hypoperfusion (55). A low PSV or reversed end diastolic flow (EDF) with raised PI has been reported in anterior cerebral artery and renal and mesenteric arteries, and the degree of resistance is proportional to the size of the shunt (56). The size of the shunt is proportional to the degree of morbidity (increased risk of intraventricular hemorrhage, NEC, acute kidney injury, and death).

The association between PDA and the risk of NEC is complex. Gut hypoxia has been demonstrated to increase the risk of NEC, and the presence of both PDA and the drugs that encourage PDA closure may cause gut hypoperfusion. Prophylactic surgical ligation may reduce the risk of NEC (57), but more recent RCTs revealed no difference in NEC rates in infants receiving prophylactic pre-symptomatic medical treatment (58). As such, prophylactic treatment of PDA is no longer used, and clinicians approach treatment more cautiously (59, 60).

McCurnin and Clyman found that the presence of a PDA limits the usual increase in post-prandial SMA flow in preterm baboons (61), and similar effects were seen in neonatal studies (55, 62, 63) (Table 4). Our group showed significantly higher PSV in preterm infants with a PDA if they were receiving at least half of the total fluids enterally compared with those who received <50%, but EDV was similar in both feed groups (37), indicating that interrupting the SMA blood flow in response to feed may impede normal digestion (62).

### Effects of congenital heart disease on SMA Doppler

Infants with left ventricular outflow obstruction of any cause (e.g., critical aortic stenosis, coarctation, or interruption of aorta) may be at risk of abnormal BFV of the SMA (reduced PSV and mean velocity and increased resistance) (28). While the ductus arteriosus is open, some blood flow is maintained to the lower extremities. However, when the duct closes, BFV in the distal vessel falls and resistance rises dramatically, causing profound hypoxia to all distal organs including the gut. This can cause a devastating, potentially fatal NEC due to a rapid and profound reduction in blood supply and oxygen delivery and causes gut ischemia.

### Other biomarkers of gut perfusion and SMA Doppler

The use of NIRS in monitoring gut oxygenation in preterm infants is still being understood. While SMA Doppler reflects blood flow, NIRS measures oxygen delivery (and subsequent oxygen extraction). These two aspects of oxygen delivery to an organ are inherently different while still being related. Nevertheless, their association is an important one to understand. Gillam-Krakauer et al. found that the change in splanchnic regional oxygenation (SrSO_2_) measured using NIRS correlated well with the changes in SMA BFV (using Doppler US) before and after feeds, suggesting that SrSO_2_ reflects intestinal blood flow and can be used to assess intestinal perfusion (64). Our group performed concurrent SMA Doppler and gut NIRS in investigating the effects of anemia and the changes in response to BT and found that BT improved intestinal oxygenation but did not alter mesenteric BFV, suggesting these two measures may be more clinically useful when used together rather than independently (37).

Lactic acidosis is a non-specific marker of tissue ischemia and is easy to identify on bedside blood test. It would be useful for future work to include SMA Doppler measurements during lactic acidosis, as well as its relationship with known biological markers of gut injury such as intestinal fatty acid binding protein (iFABP), the latter of which is currently being investigated by our group in both FGR and AGA preterm infants.

## Conclusion

SMA Doppler is a safe and reliable bedside test to assess gut blood flow for unwell and premature neonates. Doppler can assist in identifying adequate transition from fetal to newborn circulation and may be useful in high-risk infants (e.g., FGR). High resistance patterns of flow persist after birth for approximately 24 h in these infants; perhaps SMA Doppler could identify when this resistance is dropping and guide clinicians when to safely start feeding.

The risk of NEC increases with raised PI and falls with high EDV, and infants with high-risk factors for NEC have high resistance flow patterns on day 1 of life. While an early use of SMA Doppler may identify infants who are at high risk of developing NEC, there is a lack of evidence that the use of routine SMA Doppler would reduce the incidence of NEC. Clinically, SMA Doppler may be a useful tool to assess intestinal perfusion if used alongside gut NIRS.
